# Supplementary material for: Dynamic transcriptomic profiles of zebrafish gills in response to zinc supplementation
Source: BMC Genomics. 2010 Oct 11;11:553. doi: 10.1186/1471-2164-11-553 (PMC3091702; doi:10.1186/1471-2164-11-553)
Supplement: Additional file 2 — Interactive Direct Interaction Network representing the molecular interactions between zinc, copper, iron, calcium and proteins encoded by transcripts changed by zinc supplementation. Mini web-site containing index.html and hyperlinked pages in subdirectory describing a Direct Interaction Network automatically generated based on curated interactions contained within the proprietary PathwayArchitect database. Ovals represent proteins and the circles symbolize metal ions. Objects are coloured by their abundance in zebrafish at the time-point they were significantly different from the control is a scale from -4 fold (dark green) to +4 fold (dark red). Where significant differences were found at more than one time-point, the colour overlay shows expression at the first instance. Dark blue squares denote 'binding', and light blue squares 'expression'; green squares stand for 'regulation', green diamonds for 'metabolism', and green circles for 'promoter binding'. Arrow heads indicate directionality of the interaction where annotated. All nodes and edges can be further interrogated by selecting the relative area of the image. [file 1471-2164-11-553-S2.zip › PathwayArchitect Zn xs DIN/157021.html]

# PROTEIN: ZAP70

|  |  |
| --- | --- |
| Name | ZAP70 |
| Type | PROTEIN |
| Description | zeta-chain (TCR) associated protein kinase 70kDa |
| Note |  |
| Alias | AI327364 |
|  | Zap-70 |
|  | MGC108902 |
|  | ZAP-70 |
|  | zeta-chain associated protein kinase, 70kD |
|  | zeta-chain (TCR) associated protein kinase (70 kD) |
|  | TZK |
|  | syk-related tyrosine kinase |
|  | zeta-chain associated protein kinase 70kDa |
|  | 70kDa |
|  | ZAP70 |
|  | 70 kDa zeta-associated protein |
|  | Zap70 |
|  | Syk-related tyrosine kinase |
|  | zeta-chain (TCR) associated protein kinase (70kD) |
|  | truncated ZAP kinase |
|  | Srk |
|  | STD |
|  | SRK |
|  | Syk-related protein tyrosine kinase |


---

|  |  |
| --- | --- |
| GO Component | cytosol |
|  | T cell receptor complex |
|  | cytoplasm |


---

|  |  |
| --- | --- |
| GO ID | GO:0050850 |
|  | GO:0005524 |
|  | GO:0046641 |
|  | GO:0005737 |
|  | GO:0045060 |
|  | GO:0007243 |
|  | GO:0004713 |
|  | GO:0005515 |
|  | GO:0000166 |
|  | GO:0004672 |
|  | GO:0005829 |
|  | GO:0045582 |
|  | GO:0007242 |
|  | GO:0018108 |
|  | GO:0042101 |
|  | GO:0045061 |
|  | GO:0007165 |
|  | GO:0004715 |
|  | GO:0050852 |
|  | GO:0016301 |
|  | GO:0006468 |
|  | GO:0046638 |
|  | GO:0046777 |
|  | GO:0009887 |
|  | GO:0001784 |
|  | GO:0004674 |
|  | GO:0045059 |
|  | GO:0006955 |
|  | GO:0016740 |


---

|  |  |
| --- | --- |
| MIM | MIM:176947 |


---

|  |  |
| --- | --- |
| Connectivity | 436 |


---

|  |  |
| --- | --- |
| Entrez ID | 22637 |
|  | 301348 |
|  | 7535 |


---

|  |  |
| --- | --- |
| Agilent ID | A\_14\_P123690 |
|  | A\_24\_P169234 |
|  | A\_44\_P994692 |
|  | A\_51\_P327141 |
|  | A\_14\_P127563 |
|  | A\_53\_P158913 |
|  | A\_42\_P728121 |
|  | A\_53\_P173672 |
|  | A\_14\_P105304 |
|  | A\_23\_P39682 |


---

|  |  |
| --- | --- |
| Cellular Localization | Cytosol |
|  | Cytoplasm |
|  | Plasma membrane |
|  | Membrane |
|  | Cell |


---

|  |  |
| --- | --- |
| DbXref | KEGG pathway##04660##T cell receptor signaling pathway##http://www.genome.jp/dbget-bin/show\_pathway?mmu04660+22637 |


---

|  |  |
| --- | --- |
| Pathway | T-cell Receptor Activation |
|  | Zn xs inventory |
|  | Zn xs DIN |


---

|  |  |
| --- | --- |
| GO Process | immune response |
|  | positive regulation of calcium-mediated signaling |
|  | negative thymic T cell selection |
|  | autophosphorylation |
|  | peptidyl-tyrosine phosphorylation |
|  | positive thymic T cell selection |
|  | intracellular signaling cascade |
|  | protein kinase cascade |
|  | positive regulation of alpha-beta T cell differentiation |
|  | protein amino acid autophosphorylation |
|  | positive regulation of T cell differentiation |
|  | T cell receptor signaling pathway |
|  | thymic T cell selection |
|  | signal transduction |
|  | protein amino acid phosphorylation |
|  | positive regulation of alpha-beta T cell proliferation |
|  | organogenesis |


---

|  |  |
| --- | --- |
| UniGene | Rn.17862 |
|  | Hs.234569 |
|  | Mm.8038 |


---

|  |  |
| --- | --- |
| Affymetrix Probeset ID | 1393400\_at |
|  | 1394306\_at |
|  | 1422701\_at |
|  | 1439749\_at |
|  | 1440178\_x\_at |
|  | 1498\_at |
|  | 1555613\_a\_at |
|  | 214032\_at |
|  | 93661\_at |
|  | 93662\_s\_at |
|  | 93663\_r\_at |
|  | Hs2.234569.2.S1\_3p\_s\_at |
|  | Hs.234569.0.S1\_3p\_at |
|  | Hs.234569.0.S1\_3p\_x\_at |
|  | L05148\_at |
|  | Msa.282.0\_s\_at |
|  | rc\_AI012530\_at |
|  | TC34891\_at |
|  | rc\_AA955109\_at |
|  | rc\_AA955109\_g\_at |


---

|  |  |
| --- | --- |
| EC Number | EC 2.7.1.112 |


---

|  |  |
| --- | --- |
| GO Function | protein-tyrosine kinase activity |
|  | protein serine/threonine kinase activity |
|  | non-membrane spanning protein tyrosine kinase activity |
|  | transferase activity |
|  | protein kinase activity |
|  | nucleotide binding |
|  | ATP binding |
|  | protein binding |
|  | phosphotyrosine binding |
|  | kinase activity |


---

|  |  |
| --- | --- |
| Nucleotide | NM\_207519 |
|  | BC039039 |
|  | BC029727 |
|  | NM\_001012002 |
|  | AK089056 |
|  | AB083210 |
|  | AC016699 |
|  | BC053878 |
|  | AB084383 |
|  | NM\_001079 |
|  | U04379 |
|  | U77667 |
|  | NM\_009539 |
|  | AB083211 |
|  | L05148 |
|  | AL832287 |
|  | AK039883 |
|  | BC089855 |


---

|  |  |
| --- | --- |
| Protein | AAH29727 |
|  | AAH53878 |
|  | NP\_001070 |
|  | P43403 |
|  | AAH89855 |
|  | P43404 |
|  | BAC67015 |
|  | BAC43746 |
|  | NP\_033565 |
|  | AAH39039 |
|  | BAC43747 |
|  | NP\_001012002 |
|  | AAB36538 |
|  | BAC30471 |
|  | NP\_997402 |
|  | AAX93187 |
|  | AAA19250 |


---

|  |  |
| --- | --- |
| Organism | Mammal |


---

|  |  |
| --- | --- |
| Location | 1 20.5 cM (Mus musculus) |
|  | chromosome 9, 9q21 (Rattus norvegicus) |
|  | chromosome 1, 1 20.5 cM, 1 A4-C1 (Mus musculus) |
|  | chromosome 2, 2q12 (Homo sapiens) |


---

|  |  |
| --- | --- |
